# On-Board Unit (OBU)-Supported Longitudinal Driving Behavior Monitoring Using Machine Learning Approaches

**DOI:** 10.3390/s23156708

**Published:** 2023-07-27

**Authors:** Leyu Wei, Lichan Liang, Tian Lei, Xiaohong Yin, Yanyan Wang, Mingyu Gao, Yunpeng Liu

**Affiliations:** 1 School of Electronic and Information, Hangzhou Dianzi University, Hangzhou 310018, China; weileyu@cethik.com (L.W.); mackgao@hdu.edu.cn (M.G.); 2 CETHIK Group Co., Ltd., Hangzhou 314501, China; 3 College of Urban Transportation and Logistics, Shenzhen Technology University, Shenzhen 518118, China; 201904010129@stumail.sztu.edu.cn (L.L.); yinxiaohong@sztu.edu.cn (X.Y.); wangyanyan@sztu.edu.cn (Y.W.); 4 Zhejiang HIKAILINK Technology Co., Ltd., Hangzhou 311100, China; liuyunpeng@cethik.com

**Keywords:** driving behavior monitoring, machine learning, OBU, feature extraction

## Abstract

Driving behavior recognition can provide an important reference for the intelligent vehicle industry and probe vehicle-based traffic estimation. The identification of driving behavior using mobile sensing techniques such as smartphone- and vehicle-mounted terminals has gained significant attention in recent years. The present work proposed the monitoring of longitudinal driving behavior using a machine learning approach with the support of an on-board unit (OBU). Specifically, based on velocity, three-axis acceleration and three-axis angular velocity data were collected by a mobile vehicle terminal OBU; through the process of data preprocessing and feature extraction, seven machine learning algorithms, including support vector machine (SVM), random forest (RF), k-nearest neighbor algorithm (KNN), logistic regression (LR), BP neural network (BPNN), decision tree (DT), and the Naive Bayes (NB), were applied to implement the classification and monitoring of the longitudinal driving behavior of probe vehicles. The results show that the three classifiers SVM, RF and DT achieved good performances in identifying different longitudinal driving behaviors. The outcome of the present work could contribute to the fields of traffic management and traffic safety, providing important support for the realization of intelligent transport systems and the improvement of driving safety.

## 1. Introduction

With growing concern regarding the traffic congestion problem and traffic safety issues, intelligent vehicle and connected vehicle techniques have become mainstream trends in today’s automotive industry. As a result, real-time driving behavior monitoring has attracted significant attention in recent years as it can provide important reference input for intelligent vehicle control, as well as microscopic traffic estimation and control. On one hand, to support the timely response of intelligent vehicle control and ensure the safety of autonomous driving systems, it is necessary to realize driving behavior recognition for smart vehicles in real time [1]. On the other hand, dynamically monitoring the driving behavior of probe vehicles could provide fine-grained and efficient traffic information for real-time traffic estimations [2]. When recognizing driving behavior, two types of driving behavior are usually considered: is the first type includes lateral driving behaviors, such as lane changing and lane keeping, and the other includes longitudinal driving behaviors, including acceleration, braking, and stopping or cruising. Monitoring longitudinal driving behaviors can provide real-time data on the movement status of a vehicle, which is very useful for monitoring traffic flow and controlling congestion. In addition, the identification of these longitudinal driving behaviors could help identify undesirable driving behaviors, such as sudden acceleration, sharp braking, etc., so as to achieve early warnings and risk management for traffic accidents. Moreover, such information can be used to evaluate a driver’s driving style [3,4,5,6,7,8], thus helping the driver to identify and improve bad driving habits and improve driving safety.

Various approaches have been used in research for driving behavior recognition, including visual data-supported technology and motion sensing-supported technology. Approaches based on visual data technology use camera data from inside or outside the vehicle for analysis, and image processing and computer vision techniques extract key features and classify behaviors [9,10,11]. For example, some studies explored efficient methods for detecting driving behavior using visual data and obtained good performance [12,13]. Although a visual data-supported approach can obtain rich visual information, such as vehicle position, speed, and road conditions, etc., processing large amounts of unstructured video data requires extensive computation and efficient data transmission. In contrast, motion sensing-supported technology utilizes mobile sensors inside and outside the vehicle, such as accelerometers, gyroscopes, GPS, and inertial navigation systems, to obtain the vehicle’s motion state and information about its environment [14]. Through analyzing these sensing data, the features of different driving behaviors can be extracted and used for behavior recognition via machine learning approaches. Such approaches could provide real-time information for specifying driving behavior and are not limited by visual conditions. In addition, with the popularization of smartphones and in-vehicle sensing techniques, the implementation and promotion of mobile sensing technology has become more convenient.

With the development of the vehicle-infrastructure cooperative system (VICS) [15] in recent years, the OBU (on-board units) has become an essential communication device and is responsible for collecting vehicle-side information and transmitting it to external equipment. The OBU is usually embedded with acceleration sensors or IMU sensors, providing the possibility of dynamically monitoring driving behavior with future connected vehicles (CVs). Therefore, monitoring driving behavior with the support of an OBU is a promising means of collecting microscopic traffic information in ITS and could support better traffic management and safety assurance applications. However, few studies have explored the possibility of OBU-supported driving behavior monitoring.

Therefore, the present work aims to explore the possibility of applying an OBU for dynamically monitoring longitudinal driving behavior via machine learning approaches. Specifically, the real-time velocity, acceleration, angular velocity, and GPS position data of probe vehicles are collected via an OBU. Through a series of preprocessing steps, seven machine learning methods, including SVM, RF, KNN, LR, BPNN, DT, and NB, are applied to identify the longitudinal behavior of vehicles. The structure of the paper is organized as follows: Section 2 presents the data acquisition and preprocessing part of this work; Section 3 describes the applied machine learning algorithms and presents an analysis of the classification results; and finally, Section 4 summarizes and discusses the conclusions obtained in the present work.

## 2. Literature Review

In recent years, scholars have conducted extensive research on the issue of driving behavior analysis and classification. Most existing studies conducted driving behavior monitoring based on mobile sensing techniques such as smartphone- and vehicle-mounted motion sensors. For instance, some scholars used acceleration and angular velocity data collected through mobile phones and classified behaviors such as acceleration, deceleration, and turning [6,7,16,17,18]. Some scholars used in-vehicle devices embedded with IMU sensors to acquire vehicle motion data, applying machine learning methods for driving behavior monitoring [19,20,21,22,23,24]. Bonfati et al. fused the CAN bus information of a vehicle and IMU sensor information to detect the driving behavior of the vehicle [25]. As can be concluded from these studies, vehicle posture data collected through accelerometers or IMU sensors can provide important references for characterizing driving behavior.

Various classification methods have been applied to the problem of driving behavior recognition. Some research studies built specific recognition models by establishing specific rules. For instance, Bryan Higgs et al. proposed an approach for driving behavior recognition via associating known states with multiple actions [26]. Kim Schmidt attempted to detect lane change behavior by analyzing the steering wheel turning angle pattern [27]. Aside from such rule-based methods, most existing studies built behavior recognition models via machine learning approaches. Commonly used machine learning algorithms include Markov models [23,28,29,30,31,32,33], neural networks [34,35,36], SVM [37,38], etc. Dejan Mitrovic proposed the identification of driving events using the hidden Markov model (HMM) [19]. Sun H. et al. proposed an integrated behavior recognition framework with two sub-models (an anti-noise clustering algorithm and an automatic labeling method) to recognize longitudinal driving behavior with an accuracy of 92.9% [30]. Cao et al. used a car recorder’s camera and motion sensor data for driving behavior recognition based on the RF algorithm [21]. Wu et al. used a Kalman Filter to eliminate the noise of nine-axis sensor data and then applied SVM, radial basis function (RBF) network, LR, Bayesian network, C4.5 decision tree algorithm, KNN, and NB to identify different driving behaviors [22]. Some scholars applied a dynamic time warping (DTW) algorithm to detect and identify driving behaviors [16,17,39]. Other scholars applied convolutional neural networks (CNN) with multiple sources of fused data for identifying car maneuvers, and comparisons with other models such as a HMM, RF, artificial neural network (ANN), KNN, and SVM were made [24,25,26,27,28,29,30,31,32,33,34,35,36,37,38,39,40]. Junior et al. used four algorithms, including multilayer perceptron (MLP), SVM, RF and Bayesian network, to identify driving behaviors such as fast lane changes, turns, braking, and acceleration, and the results showed that RF and MLP demonstrated better performances [18]. Al-Din and Najim, M.S. used RF, SVM, and KNN to identify and classify the acceleration, deceleration, and lane changes of vehicles and found that three parameters, longitudinal acceleration, lateral acceleration, and the yaw angle, were sufficient to identify and classify driving maneuvers [41]. As can be concluded from the existing studies, machine learning-based methods have achieved good performance in driving behavior recognition, while the accuracy and robustness of these methods applied to real-time longitudinal driving behavior monitoring still merit further exploration.

While specifying longitudinal driving behavior recognition, Lyu used longitudinal driving behavior data to annotate longitudinal driving operating conditions (DOCs) and identified driving styles based on these DOCs and machine learning classifications, which informed real-time online driving style recognition for vehicles equipped with in-vehicle data acquisition devices [42]. Lei Yang proposed a new deep belief network (DBN) to predict the front wheel angle and speed of a vehicle through combining a multi-target sigmoid regression (MSR) layer with a DBN, called MSR-DBN, to optimize the prediction of lateral and longitudinal behaviors. The prediction results of the MSR-DBN were compared with those of general DBN model, back-propagation (BP) neural network, SVR, and RBF network. The results show that the proposed MSR-DBN outperformed the other models in terms of accuracy and robustness [43]. These studies further examined the effectiveness of applying machine learning approaches in longitudinal behavior recognition. Based on the above considerations, this paper therefore takes an OBU with embedded IMU sensor as basic data acquisition unit and intends to realize accurate perception of longitudinal driving behavior through effective data mining and machine learning algorithms so as to verify the feasibility of OBU-supported driving behavior recognition and to provide an effective solution for micro-behavior perception in the VICS environment.

## 3. Data Collection and Preprocessing

**Figure 1:**
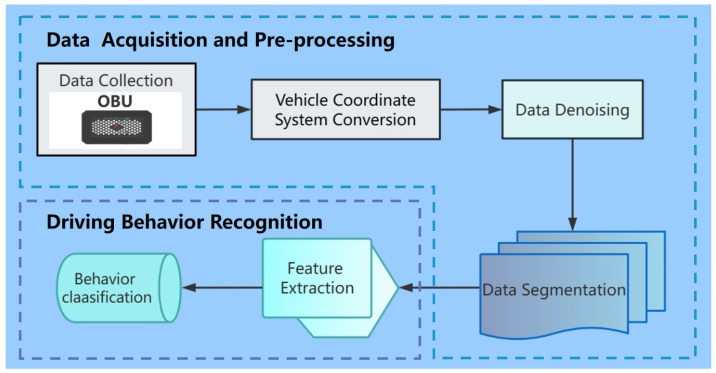
Implementation Process.

The implementation process of the whole system proposed in the present work can be summarized in two major parts: data acquisition and preprocessing and driving behavior recognition. In the data acquisition and preprocessing part, the required driving data are first collected via embedded IMU and GPS sensors in an OBU. The collected data are then preprocessed through three major steps: vehicle coordinate system conversion (VCSC), data denoising, and data segmentation. These preprocessing steps aim to provide high-quality data support, facilitating the subsequent recognition of longitudinal driving behavior. During behavior recognition, longitudinal driving behavior is recognized by extracting the segmented data features and inputting them into the behavior classification model for training. The implementation process is shown in Figure 1.

### 3.1. Data Collection

**Figure 2:**
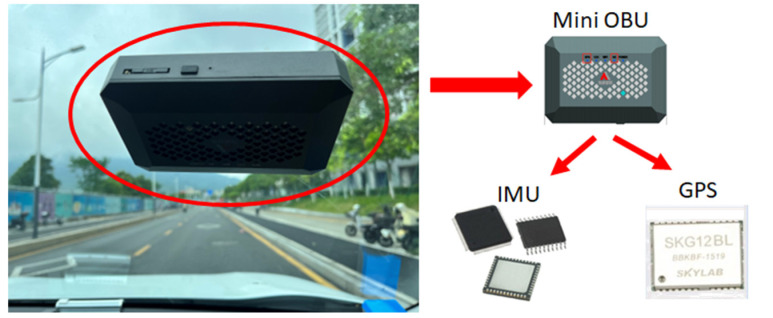
Equipment Installation.

In the present work, real-time driving data were collected via a field experiment. The equipment used for data collection was a Mini OBU (model number: HKZL-CVI-OH001) developed by Zhejiang HIKAILINK Technology Co., Ltd., Hangzhou, China. The MINI OBU is a compact and easy-to-install intelligent vehicle product with both ETC (electronic toll collection) and V2X (vehicle to everything) functions, which can support ETC functions and form an intelligent vehicle terminal with V2X, high-precision positioning, and ETC as its core. OBU can be simply deployed in the vehicle and has is compact in size. It can integrate some of the existing functions of current on-board equipment inside vehicles, which can help rapidly popularize vehicle–road cooperation from the vehicle side and further improve the C-V2X user loading rate. It has a sampling frequency of 10 Hz. The OBU is embedded with an inertial measurement unit (IMU) and GPS. The IMU facilitates the acquisition of three-axis vibration pulse data, while the GPS enables real-time recording of the vehicle’s longitude and latitude position information. During the test, the OBU was securely installed on the vehicle’s front windshield, as illustrated in Figure 2.

**Figure 3:**
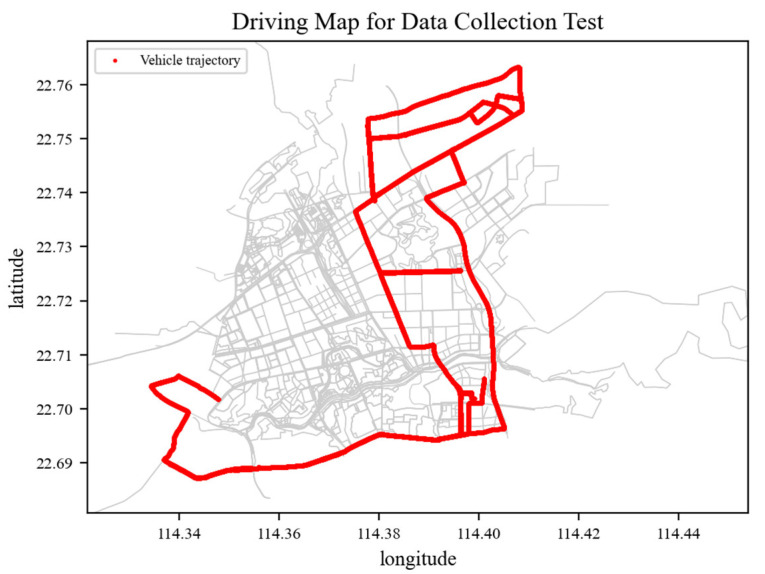
Driving map for data collection test.

The data collection experiment was conducted on sections of an urban road within the Pingshan District in Shenzhen, as presented in Figure 3. Specifically, the vehicle speed, acceleration, angular velocity, latitude, longitude position, and other relevant parameters were collected during the data collection test. Table 1 demonstrates the data export parameters and their format. Video recording was conducted using cell phones during the experiment to capture the actual driving behavior and the associated traffic condition, providing a basis for further validation of the driving behavior classification results. Statistically, we collected a total of 93,866 data sets.

### 3.2. Vehicle Coordinate System Conversion (VCSC)

**Figure 4:**
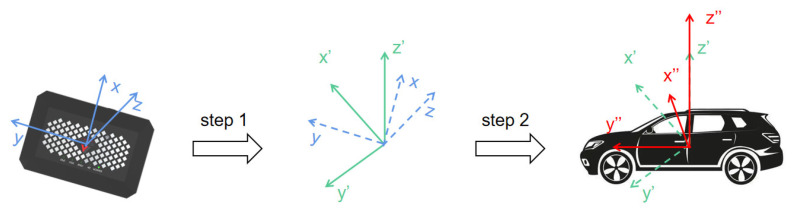
Process of VCSC.

Since the fixed orientation of the accelerometer in an OBU differs from the actual orientation of the vehicle, vehicle coordinate system conversion (VCSC) should be performed to obtain the actual acceleration in the vehicle’s coordinates [44,45,46]. The default coordinate system of the built-in three-axis accelerometer in the OBU and its acceleration inertial forces are illustrated in Figure 4. As the coordination of the built-in accelerometer in an OBU is not clear, it is critical to convert the recorded acceleration into the actual acceleration within the vehicle’s reference attitude via the coordinate system conversion step. In the present work, the VCSC step was carried out using Euler angle conversion. The Euler angles encompass the roll angle *α*, pitch angle *β*, and yaw angle *γ*. To effectuate this transformation, the sensor coordinate system was subjected to sequential rotations of *α*, *β*, and *γ* around the *x*-axis, *y*-axis, and *z*-axis, respectively, as per Equation (1). The roll angle *α* is in the range of [−π; π], the pitch angle *β* is in the range of [−π/2; π/2], and the yaw angle *γ* is in the range of [−π; π].

(1) $$\left[\begin{array}{c} a_{x}^{\prime \prime }\\ a_{y}^{\prime \prime }\\ a_{z}^{\prime \prime } \end{array}\right]=\left[\begin{array}{ccc} \cos\gamma & -\sin\gamma & 0\\ \sin\gamma & \cos\gamma & 0\\ 0 & 0 & 1 \end{array}\right]\left[\begin{array}{ccc} \cos\beta & 0 & \sin\beta \\ 0 & 1 & 0\\ -\sin\beta & 0 & \cos\beta \end{array}\right]\left[\begin{array}{ccc} 1 & 0 & 0\\ 0 & \cos\alpha & -\sin\alpha \\ 0 & \sin\alpha & \cos\alpha \end{array}\right]\left[\begin{array}{c} a_{x}\\ a_{y}\\ a_{z} \end{array}\right]$$

The implementation of VCSC can be divided into two distinct steps [47]. The first step of VCSC is to align the *z*-axis of the device coordinate system with the *z*-axis of the car coordinate system using the vehicle’s gravity property, as depicted in Figure 3. This process first employs a sliding window approach to extract a 6 s data window of the *z*-axis at 2 s intervals. The standard deviation of the data within each window is then calculated. By identifying the window with the lowest standard deviation, the most stable window is determined. The average value of the three axes within the selected window is then used to compute the cross-roll angle *α* and pitch angle *β* using Equations (2) and (3) [48]. Ultimately, the first step of the conversion is accomplished by applying Equation (4).

(2) $$\alpha =\tan^{-1}\left(\frac{a_{y}}{a_{z}}\right)$$

(3) $$\beta =\tan^{-1}\left(\frac{-a_{x}}{\sqrt{a_{y}^{2}+a_{z}^{2}}}\right)$$

(4) $$\left[\begin{array}{c} a_{x}^{\prime }\\ a_{y}^{\prime }\\ a_{z}^{\prime } \end{array}\right]=\left[\begin{array}{ccc} \cos\beta & 0 & \sin\beta \\ 0 & 1 & 0\\ -\sin\beta & 0 & \cos\beta \end{array}\right]\left[\begin{array}{ccc} 1 & 0 & 0\\ 0 & \cos\alpha & -\sin\alpha \\ 0 & \sin\alpha & \cos\alpha \end{array}\right]\left[\begin{array}{c} a_{x}\\ a_{y}\\ a_{z} \end{array}\right]$$

The second step of the VCSC process is to align the *x*-axis and *y*-axis of the sensor coordinate with the vehicle coordinate system based on braking or acceleration events. This specific process involves using a sliding window approach with 2 s intervals to extract 6 s windows. For each window, ∆v (change in velocity) and $∆z^{\prime }$ (change in acceleration in the *z*-axis) are calculated. Windows that satisfy the conditions ∆v>5, $∆z^{\prime }<1.5$, and $∆v-∆z^{\prime }>4$ are considered a single event. The most representative window is selected based on the criterion that $∆v-∆z^{\prime }$ is the largest. The angle *γ* is then calculated using Equation (5) [48]. Finally, the first step of the conversion is completed by applying Equation (6) [48].

(5) $$\gamma =\tan^{-1}\left(\frac{a_{x}^{\prime }}{a_{y}^{\prime }}\right)$$

(6) $$\left[\begin{array}{c} a_{x}^{\prime \prime }\\ a_{y}^{\prime \prime }\\ a_{z}^{\prime \prime } \end{array}\right]=\left[\begin{array}{ccc} \cos\gamma & -\sin\gamma & 0\\ \sin\gamma & \cos\gamma & 0\\ 0 & 0 & 1 \end{array}\right]\left[\begin{array}{c} a_{x}^{\prime }\\ a_{y}^{\prime }\\ a_{z}^{\prime } \end{array}\right]$$

**Figure 5:**
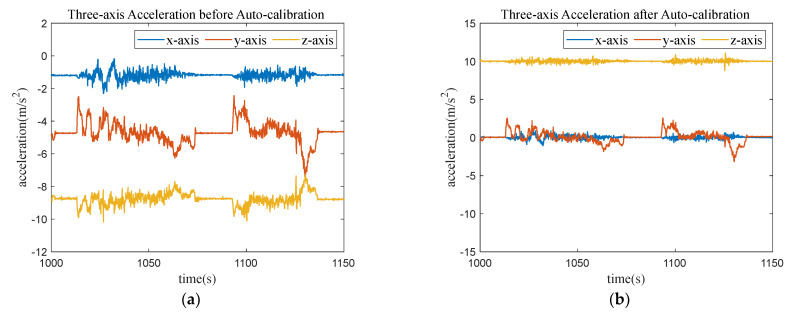
Comparison of acceleration before and after VCSC. (**a**) Acceleration before VCSC; (**b**) Acceleration after VCSC.

The effect of VCSC can be observed in Figure 5, in which Figure 5a depicts the OBU device’s coordinate system data before VCSC, and Figure 5b illustrates the converted acceleration in vehicle coordinates after the VCSC step.

As the sensor device used in the experiment was fixed on the vehicle, the angle can be calculated and applied to all the collected data. Generally, the VCSC process is conducted 5 min after the device is turned on to ensure the reliability of the results.

### 3.3. Data Denoising

When it comes to data denoising, commonly employed methods include Kalman filtering [49,50] and low-pass filtering. These techniques are frequently utilized to enhance the quality of data by reducing noise and unwanted disturbances. The application of these approaches plays a crucial role in improving the accuracy and reliability of the data for subsequent analysis and processing tasks. Low-pass filtering is a widely used method for signal filtering. It follows a principle in which low-frequency signals are allowed to pass through, while high-frequency signals that surpass a predetermined threshold are obstructed and diminished. Nevertheless, the extent of the obstruction and diminishment varies based on distinct frequencies and filtering procedures, serving different purposes. The first-order low-pass filtering approach incorporates the current sample value and the preceding filtered output value to establish the practical filter value, thereby introducing a feedback effect on the input to achieve the desired output.

**Figure 6:**
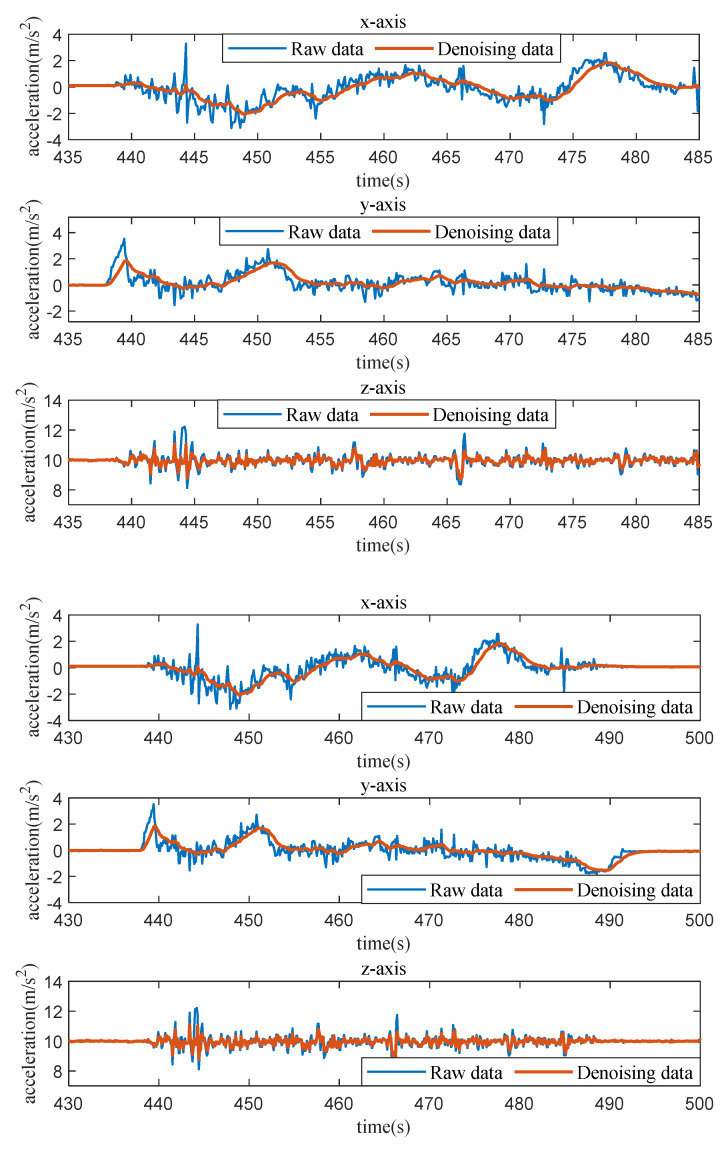
The effect of data denoising.

Due to sensor errors and the impact of various scenarios, the acquired vehicle acceleration data tend to be noisy, exhibiting numerous fluctuations within the acceleration curve and thereby posing challenges for feature extraction. In this study, low-pass filtering is employed as a means of filtering and smoothing the vehicle acceleration data, reducing efficient noise while preserving the inherent features of the data without any distortion. The results of the three directions are depicted in Figure 6, wherein the original vehicle acceleration data are represented by the blue line, and the low-pass-filtered acceleration data are represented by the red line.

### 3.4. Data Segmentation

The road traffic conditions change during driving, resulting in various driving behaviors. Consequently, the time duration associated with each driving behavior often lacks consistency. Therefore, a data segmentation step is usually needed to first separate the time series data into segments such that each segment corresponds a specific driving behavior. Although a fixed-time-window segmentation method could be applied, it may lead to the loss of valid data and a lower detection accuracy. This highlights the challenge of accurately extracting segments of driving behavior. One approach to address this challenge is the detection of behavior change points, which serves as a method for extracting driving behavior segments. Currently, many researchers utilize template matching algorithms and change point detection algorithms to identify and analyze driving behaviors. Among these approaches, the change point detection algorithm proves to be more efficient than the template matching algorithm. Moreover, it eliminates the need for a predefined standard driving behavior template.

The present work therefore adopted a change point detection algorithm to solve the data segmentation problem. Specifically, the average energy method (AEM), which was initially developed for solving the speech endpoint detection problem, was applied. The AEM is usually used to identify the starting and ending points of speech by analyzing the short-term characteristics of the human voice signal [51,52], offering the benefits of straightforward computation and intuitive interpretation. In the present work, the time-varying acceleration data during driving could be taken as time-series signal data, and thus the AEM can be applied to solve the data segmentation problem by dynamically analyzing the short-term characteristics of the acceleration signal.

Taking the *y*-axis acceleration data as an example, a fundamental time window is established to compute the average energy of the data within that window. The calculation formula for determining the average energy is as follows:

(7) $$\bar{E}=\frac{g_{y}^{2}(i)+g_{y}^{2}(i-1)+\ldots +g_{y}^{2}(i-k-1)}{k}$$

where $g_{y}(i)$ represents the acceleration of point i; and k represents the length of the time window.

Taking the acceleration data of the *y*-axis as an example, a baseline time window is set, and the average energy of the data is calculated within the time window. The calculation formula is as follows:

① The selection of the threshold value has a significant influence on the detection of behavior change points. By analyzing the basic data and average energy, the threshold value $E_{T}$ is set to 0.1. The maximum sampling time is set to 12 s, and the minimum sampling time is 1 s, i.e., the maximum number of sampling points $L_{\max}$ = 120 and the minimum number of sampling points $L_{\min}$ = 10. Additionally, a behavior change location matrix is established in which the starting point of the behavior is recorded in the first column and the end point of the behavior is recorded in the second column.

② The average energy $\bar{E}(i)$ between the sampling points i and i−k+1 is calculated, and when $\bar{E}(i)\geq E_{T}$ is satisfied, the sampling points i−k+1 are deposited in the first column of the position matrix as the starting point of the behavior.

③ For i=i+1 in turn, the average energy value is calculated and compared with the set threshold $E_{T}$. If $\bar{E}(i)<E_{T}$, the sampling point i−k+1 is stored in the second column of the position matrix, which is the end point of the behavior.

④ If the continuous sampling point is equal to the maximum number of sampling points, the point is set as the identified end point, and to the process moves to step ② again.

**Figure 7:**
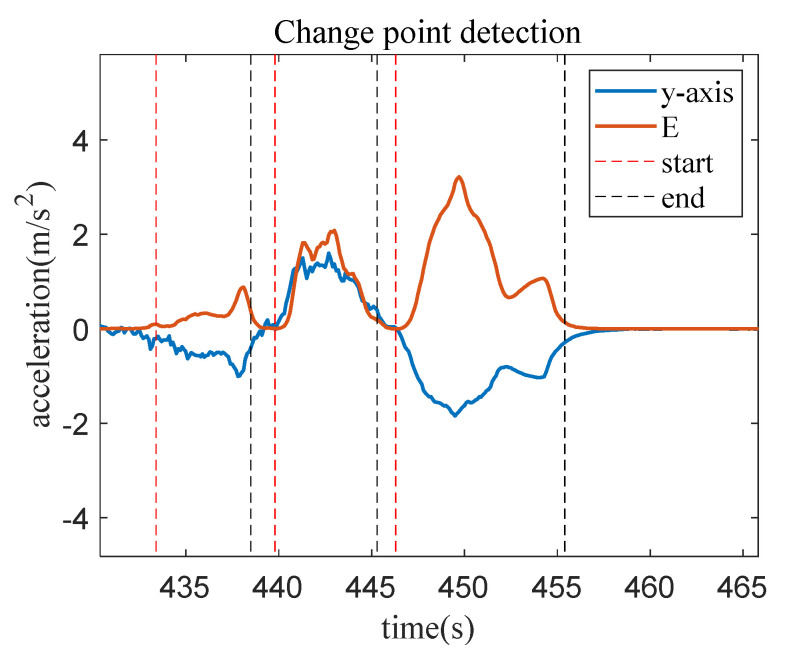
Data segmentation results through AEM.

The results of extracting the switching points for the *y*-axis acceleration during driving are depicted in Figure 7. The blue solid line represents the *y*-axis acceleration data, while the red solid line represents the average energy value. The vertical lines in the figure indicate the switching points for behavior changes, with the red vertical line indicating the start of the behavior and the black vertical line indicating the end of behavior recognition. At the beginning of the accelerated driving behavior, the average energy value is approximately zero. As longitudinal acceleration is initiated, the *y*-axis acceleration undergoes changes, and the average energy value gradually increases. When the accelerating behavior approaches to the end, the average energy value gradually returns to zero. Throughout the duration of the accelerating behavior, the average energy value generally exhibits a trend of first increasing and then decreasing. The change pattern of the average energy value for deceleration behavior is opposite to that of acceleration behavior, exhibiting a trend of first decreasing and then increasing.

### 3.5. Feature Extraction

**Figure 8:**
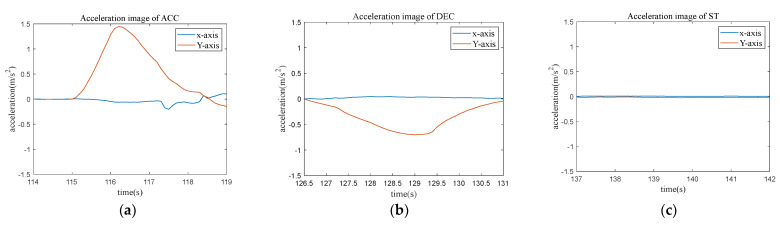
Characteristics of acceleration for different driving behaviors. (**a**) Characteristics of ACC; (**b**) characteristics of DEC; (**c**) characteristics of ST.

Feature extraction is a critical step for driving behavior recognition which aims to extract the most relevant information used for the driving behavior recognition step. In the present work, we mainly extracted relevant features from the data considering the requirement of reliability and independence between different features. This study focused on three longitudinal behaviors: acceleration, deceleration, and stopping, and the characteristics of the vehicle’s acceleration for these behaviors are illustrated in Figure 8. Through the driving test, we have observed 616 times of acceleration (ACC) behavior, 580 times of deceleration (DEC) behavior and 145 times of stopping (ST) behavior in total. It should be noted that the actual driving behavior was labeled based on a video recording made during the driving test, which was used for further validation. Through a comprehensive analysis of the characteristics for each behavior, fifteen data features were extracted, and their descriptions are provided in Table 2.

The selected features include both dimensional and dimensionless eigenvalues. The dimensional eigenvalues encompass various measures such as the maximum value, minimum value, peak-to-peak value, mean value, variance, mean square value, and root mean square value. These dimensional eigenvalues can vary in magnitude due to changes in external physical quantities. In addition to the dimensional eigenvalues, our work also incorporated a range of dimensionless eigenvalues. These dimensionless eigenvalues include the peak, pulse, margin, waveform, skewness, and cliffs.

The peak factor represents the extremity of the peak in the waveform. The pulse factor is the ratio of the signal’s peak value to the rectification’s average value. The peak factor and pulse factor are indicators used to detect the presence or absence of shocks in the signal. The margin factor is the peak/square root amplitude of the signal. The waveform factor is the ratio of rms to the rectified mean.

Skewness, also referred to as skew, is a measure of the direction and extent of asymmetry in a statistical data distribution. It quantifies the degree to which the data are skewed to the left or right of the mean. Kurtosis, also known as cliffs, measures the flatness or peakedness of a waveform and describes the distribution of a variable. Skewness and kurtosis are related to each other. Kurtosis is the ratio of the fourth-order central moment to the fourth-order standard deviation, while skewness is the ratio of the third-order central moment to the third-order standard deviation. Both skewness and kurtosis provide information about the shape of the data distribution.

## 4. Driving Behavior Recognition

In this section, we applied seven machine learning algorithms, including SVM, LR, KNN, BPNN, RF, DT, and NB. These algorithms were chosen to provide a diverse set of classification approaches to compare and evaluate their performance in recognizing different driving behaviors.

### 4.1. Algorithm Introduction

The supervised learning algorithms adopted in the present study included the SVM, RF, KNN, LR, BPNN, DT, and NB algorithms.

SVM: SVM is a binary classification model that is based on a linear classifier defined in the highest-dimensional intervals across the feature space. It assumes that all training data are linearly separable. However, the nonlinear SVM algorithm is more commonly used for classification tasks. The principle behind using kernel functions is to transform the input nonlinear problem into a linear problem in a feature space with potentially higher dimensions. By raising the dimensions, linear support vector machines can effectively operate in high-dimensional spaces. Overall, the support vector machine is a classification method that relies on a small sample to make changes in the classification of the sample easier.

RF: An RF is a parallel integrated supervised learning method. An RF builds an integrated learner with the decision tree algorithm as the base learner and further introduces random attribute selection in the training of the decision tree. In an RF, a subset containing k attributes is selected from the set of attributes of each node of the base decision tree, and then the optimal attributes are selected from this subset to divide the data that can effectively filter the feature values of the data. Here, the parameter k controls the size of the randomness. The advantage of the RF algorithm is that it is simple and easy to implement while maintaining a very efficient performance and more data constituting more basic decision trees. Moreover, random forests can usually converge to a much lower generalization error.

KNN: The KNN algorithm is a simple and mature supervised learning method that is not controlled by parameters and exhibits inertia. However, it bears some similarities to the unsupervised learning algorithm known as K-means. In KNN, when predicting a new value, the category to which it belongs is determined based on the categories of the K nearest points. The KNN algorithm is well-suited for classification tasks with large sample sizes and low levels of complexity. It is considered a lazy learning algorithm as it does not construct an explicit model during the training phase.

LF: The LF assumes that the data follow a Bernoulli distribution and employs a gradient descent to estimate the parameters using the maximum likelihood function. This approach enables the classification of data into two categories.

BPNN: A BPNN consists of a multilayer neural network which connects the input and output layers by connecting a large number of neurons, and the training set is used as the input layer by setting a certain number of hidden layer neurons. Among them, the number of hidden units and layers control the weight and complexity of the whole neural network work. Too few hidden units will lead to underfitting, and too many hidden units will lead to overfitting, which makes it difficult to achieve the desired results. Therefore, it is crucial to set the right number of hidden units.

DT: A DT is a supervised learning classification algorithm. A decision tree is based on a tree structure which first generates a random number of training sets, makes a series of judgments or “sub-decisions” for the problem requiring a decision, and finally outputs the required decision. Typically, a decision tree contains a root node, numerous internal nodes, and several leaf nodes. The leaf nodes correspond to the decision results, the other nodes correspond to the attribute tests, and the root node corresponds to the final full set of samples. In the process of learning to classify a decision tree, in order to classify the samples as correctly as possible, the entire decision tree system makes too many sub-decisions, i.e., too many nodes to result in overfitting. In this case, certain branches must actively be eliminated via pruning to reduce the risk of overfitting.

NB: A classification method based on Bayes’ theorem and the assumption of the independence of characteristic conditions. The basic assumption of the NB method is that each feature is independent and equal. That is, each feature is independent with each other and each feature affects the outcome to the same degree. It is a classification method with simple logic and less storage space required for classification.

### 4.2. Classifier Evaluation

The typical performance metrics of two classification models take four types of classification accuracy: accuracy, precision, recall, and an F1-score.

Nevertheless, in our work, a triple classification of longitudinal driving behavior was used. Unlike the model performance evaluation metrics of the two-classification model, the performance metrics of the three-classification model use classification accuracy. The calculation of the classification accuracy is shown in Equation (8).

(8) $$Accuracy=\frac{T}{T+F}$$

In Equation (8), the specific meanings of *T* and *F* are shown in Table 3.

In general, the higher the accuracy, the better the classifier’s effect.

### 4.3. Results and Analysis

In this section, seven classification algorithms were applied to classify and recognize these three types of driving behaviors. The dataset was split into a training set comprising 70% of the data and a test set comprising the remaining 30%. To mitigate data dependence, 4-fold cross-validation was employed. The performance of classification results are shown in Table 4.

As can be seen in Table 4, the SVM, RF, LR, and DT algorithms all achieved very good classification performances in longitudinal driving behavior recognition, with an overall accuracy of over 97%. Specifically, the classification results of the longitudinal driving behavior of each model are shown in Table 5. As a comparison, the performances of KNN and NB are not as good as the others. The potential reasons for this could be that the ST behavior sample size is relatively small, KNN relies on neighboring samples for classification, and NB may have been affected by imbalanced data when calculating prior probabilities. In article [22], the SVM method achieved the highest accuracy of 93.25% in correctly identifying driving behaviors, demonstrating the best performance. The Bayes Net method followed closely, with an accuracy of 91.1%, while the LR method achieved an accuracy of 89.3% in recognizing driving behaviors. On the other hand, the RBF network, DT, NB, and KNN methods exhibited relatively poorer performances with accuracies of 84.75%, 83.2%, and 82.95%, respectively. However, in our study, the lowest accuracy achieved was 0.938, indicating that the proposed method effectively monitors and recognizes longitudinal driving behaviors.

**Figure 9:**
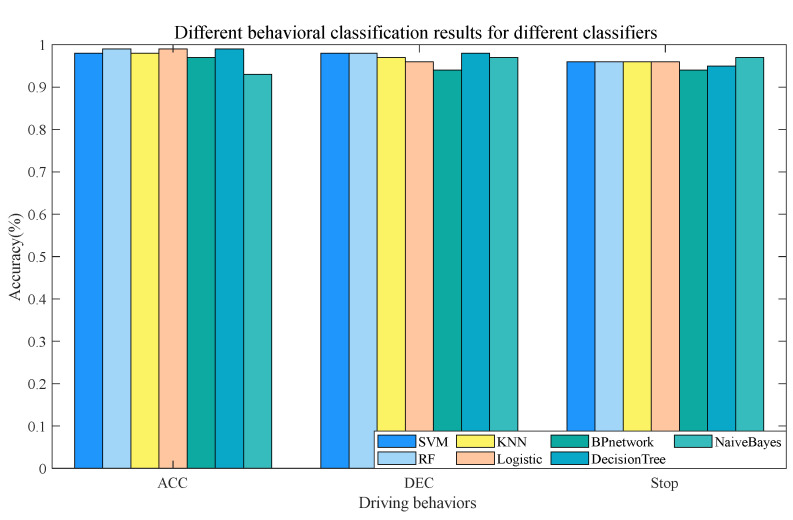
Results of predicting different driving behaviors using different algorithms.

Figure 9 shows the results of predicting different driving behaviors using different algorithms. All algorithms showed good classification performances for “ACC” behavior, with an accuracy of above 97% except for NB, which also exceeds 90%. The accuracy of all algorithms for “DEC” behavior is around 95% except for NB, which also exceeds 90%. The overall recognition of “ST” behavior is lower than the first two behaviors, especially for the BP neural network algorithm, with an accuracy rate below 95%.

An analysis of the above results shows that these seven algorithms achieved reasonably high accuracies for the test set, as the “ACC” and “DEC” samples comprised most of the data set. In contrast, the total sample size of “ST” is less than 200, which is too small; thus, the classification results are not satisfactory. The RF, SVM, and decision tree algorithms were more effective. The RF obtained a relatively high accuracy on the training set because its structure is more complex than other classifiers. The SVM had the highest accuracy for “DEC”, although it did not perform as well as the RF and decision tree for “ST”. The decision tree achieved good accuracy in the recognition of “ACC” and “DEC” and had the highest accuracy in the recognition of “ST”, which indicates its low computational power requirement and good performance. It is suitable for cases with low detection requirements and poor data quality.

In Table 5, we can see that more ST behaviors are misclassified as ACC behaviors, and a brief analysis was performed to address this phenomenon. There are two possible reasons for this situation. First, the sample size of the ST behaviors is too small and will have a high probability of random error. Second, the ACC behavior occurs before the ST behavior, and most of the ST behaviors in this experiment occurred at the traffic light position, where the acceleration is slow when the car starts.

## 5. Discussion and Conclusions

The present work explored the possibility of applying an OBU for dynamically monitoring longitudinal driving behavior via machine learning approaches. Firstly, the real-time velocity, acceleration, angular velocity, and GPS position data of probe vehicles were collected using IMU sensors embedded in an OBU. Considering the coordination difference between the vehicle and the sensors in an OBU, an auto-calibration of the vehicle’s acceleration was first conducted to obtain the three-axis acceleration of the probe vehicle. After denoising data using a low-pass filter, a change point detection model based on the average energy method was developed to realize data segmentation for further behavior classification. Using 18 extracted features, seven machine learning methods, including SVM, RF, KNN, LR, BPNN, DT, and NB, were applied to identify the longitudinal behavior of vehicles, and the performances of these methods were evaluated.

As can be concluded in the present work, all the classifiers achieved good performances, indicating that the preprocessing steps and feature extraction step work well for ensuring data quality. Based on the results, three out of the seven classifiers, SVM, RF, and DT, obtained high accuracies while classifying longitudinal driving behavior. Even the lowest accuracy achieved by NB exceeded 90%. Compared with the results obtained in some previous works [22,53], most classifiers applied in the present work achieved better performances. Although some studies seem to achieve better accuracy than the present work, for instance, Gurdit Singh [17] utilized the DTW model for detecting deceleration behaviors, yielding a perfect accuracy rate of 100% when detecting and recognizing deceleration braking behaviors, such a high degree of accuracy is aligned with a high computational complexity and lacks a clear mathematical explanation. As a comparison, the machine learning methods adopted in our work achieved good accuracies with relatively higher computational efficiencies. In addition, the models established in the present work are more optimized in terms of feature extraction, which enables them to better capture the key features of driving behavior.

Although the adopted methods obtained good performances in longitudinal driving behavior recognition, future work could be considered in improving the performances of different classifiers by optimizing the feature selection steps. Also, attention could be paid to improving the classification efficiency so that the driving behavior monitoring function can work on board the OBU terminals in real time. Furthermore, the proposed system and method in horizontal driving behavior recognition could be applied in future work.

## Figures and Tables

**Table 1:** Parameters and format of data exported by OBU.

Data Variable	Unit	Description	Example Data
position_3d.lat	-	Latitude	22.695279
position_3d.lon	-	Longitude	114.380882
sensor_3d.x_accel	m/s^2^	Acceleration in *X*-axis	−5.414062
sensor_3d.x_rotat	deg/s	*X*-axis angular velocity	−0.807861
sensor_3d.y_accel	m/s^2^	Acceleration in *Y*-axis	−1.543945
sensor_3d.y_rotat	deg/s	*Y*-axis angular velocity	0.358154
sensor_3d.z_accel	m/s^2^	Acceleration in *Z*-axis	−8.397461
sensor_3d.z_rotat	deg/s	*Z*-axis angular velocity	0.251465
motion. heading	deg	Angle of orientation	285.29
motion. speed	m/s	Speed	4.831148
Update Timestamp	-	Time of data collection	2022-07-16T01:26:57.581Z

**Table 2:** Extracted features.

Feature	Description
max	Maximum value of the data unit
min	Minimum value of the data unit
rms	Root mean square of the data unit
std	Mean squared deviation of the data unit
energy	Energy of the data unit
peak	Peak to peak value of the data unit
avg	Rectified average value of the data unit
sk	Skewness of the data unit
ku	Kurtosis of the data unit
S	Waveform factor of the data unit
L	Margin factor of the data unit
C	Peak factor of the data unit
I	Pulse factor of the data unit
k	Slope of the data unit
mean	Mean of the data unit

**Table 3:** Three-category confusion matrix.

Class	0	1	2
0	T_00_	F_01_	F_02_
1	F_10_	T_11_	F_12_
2	F_20_	F_21_	T_22_

**Table 4:** Results of different classifiers.

Model	Accuracy for Training Set	Accuracy for Testing Set
SVM	0.989	0.970
RF	0.989	0.982
KNN	0.96	0.923
LR	0.991	0.970
BPNN	0.989	0.942
DT	0.993	0.976
NB	0.938	0.915

**Table 5:** The classification results of specific driving behaviors.

Model	Behavior Category			
SVM		ACC	DEC	ST
	ACC	609	5	2
	DEC	7	570	3
	ST	3	2	140
RF		ACC	DEC	ST
	ACC	612	3	1
	DEC	8	572	0
	ST	2	3	140
KNN		ACC	DEC	ST
	ACC	607	7	2
	DEC	12	563	5
	ST	2	3	140
LR		ACC	DEC	ST
	ACC	610	5	1
	DEC	7	571	2
	ST	4	1	140
BPNN		ACC	DEC	ST
	ACC	603	6	7
	DEC	8	562	10
	ST	5	3	127
DT		ACC	DEC	ST
	ACC	614	2	0
	DEC	7	573	0
	ST	4	2	139
NB		ACC	DEC	ST
	ACC	570	21	21
	DEC	8	537	35
	ST	0	3	142

## Data Availability

The data is contained within the article.
